# In this month’s Bulletin

**DOI:** 10.2471/BLT.16.001016

**Published:** 2016-10-01

In the editorial section, Alana Officer et al. (710) highlight the hidden costs of aged-based discrimination against people. Andrew D Haddow & John P Woodall (711) emphasise a distinction between Zika and Spondweni viruses. 

Dale Gavlak (714–715) reports on local training for mental health professionals in the eastern Mediterranean region. Epidemiologist Rosa Moreira tells Andréia Azevedo Soares (716–717) how Angola managed to rein in its worst yellow fever epidemic in 30 years. 

## Bangladesh, Ghana, India, Pakistan, the United Republic of Tanzania & Zambia 

### Surviving the first day of life

Abdullah H Baqui et al. (752–758) use trial data to estimate neonatal mortality.

## Belgium, Luxembourg & the Netherlands

### Overcoming obstacles to treatment 

Séverine Henrard & Francis Arickx (779–781) describe how joint price negotiations can apply to drugs for rare diseases. 

## Brazil

### Introducing transgenic *Aedes aegypti*

Paulo Paes de Andrade et al. (766–771) examine the risk assessment done for a new method of mosquito control. 

## Cambodia

### Getting reliable results from lab tests

Lucy A Perrone et al. (743–751) describe a quality-improvement training programme for laboratory technicians. 

## China 

### Ensuring adequate food and water

Shilu Tong et al. (759–765) describe the population health implications of climate change.

## India 

### Leveraging contact with the health system

Mira Johri et al. (718–727) study the feasibility of providing additional child health services during measles vaccination campaigns. 

### How many covered?

Pankaj Bhatnagar et al. (728–734) estimate the proportion of children receiving vaccines. 

### Silicosis in mining and construction workers 

Nandini Sharma et al. (777–778) propose a comprehensive approach to prevention, recognition, and treatment of this occupational disease.

## Malawi

### Treating HIV at a national level

Andreas Jahn et al. (772–776) recount the steps taken to scale-up antiretroviral therapy.

## Global

### Ensuring access to chemotherapy

Jane Robertson et al. (735–742) compare essential medicine lists for cancer treatment.

### When direct evidence is lacking

Steve Kanters et al. (782–784) describe how network meta-analysis can be used in clinical guidelines.

